# Editorial: Microbial interaction with banana: mechanisms, symbiosis, and integrated diseases control

**DOI:** 10.3389/fmicb.2024.1390969

**Published:** 2024-04-05

**Authors:** Si-Jun Zheng, Huigang Hu, Yunfeng Li, Jian Chen, Xundong Li, Tingting Bai

**Affiliations:** ^1^Yunnan Key Laboratory of Green Prevention and Control of Agricultural Transboundary Pests, The Ministry of Agriculture and Rural Affairs International Joint Research Centre for Agriculture, The Ministry of Agriculture and Rural Affairs Key Laboratory for Prevention and Control of Biological Invasions, Agricultural Environment and Resource Research Institute, Yunnan Academy of Agricultural Sciences, Kunming, Yunnan, China; ^2^Bioversity International, Kunming, Yunnan, China; ^3^Key Laboratory of Tropical Fruit Biology, Ministry of Agriculture, South Subtropical Crop Research Institute, Chinese Academy of Tropical Agricultural Science, Zhanjiang, China; ^4^College of Plant Protection, South China Agricultural University, Guangzhou, China; ^5^College of Food Science and Technology, Hainan University, Haikou, China

**Keywords:** banana, biocontrol, disease suppression, diversity, Fusarium wilt of banana, microbiome, Tropical Race 4

## 1 Introduction

Bananas serve as important fruits globally and as the main food source in developing countries. Because of their vegetative propagation, they are vulnerable and susceptible to disease. Therefore, new integrated protection technologies are urgently needed for sustainable production. The highly destructive soil-borne fungal pathogen that triggers Fusarium wilt of bananas (FWB) is *Fusarium oxysporum* f. sp. *cubense* (*Foc*), especially Tropical Race 4 (TR4). Effective detection and validation are important before implementing adequate management (Yang et al., 2023). Comprehensive approaches to the prevention and management of existing and emerging diseases are critical for maintaining a healthy banana industry (Xu et al., 2011).

Currently, climate change has dramatically resulted in yield loss caused by shifting to pests and diseases (Bebber, 2019; Varma and Bebber, 2019; Alkhalifah et al., 2023; Kilwenge et al., 2023) and emerging diseases, such as banana blood disease (Remenant et al., 2011; Ray et al., 2021; Prakoso et al., 2022; Rincón-Flórez et al., 2022). Biocontrol agents play important roles in addressing these challenges.

## 2 Content of Research Topic

This topic has arisen from increasing awareness that microbes play an important role in mitigating soil-borne diseases. Original research articles on six aspects were collected: cover plants in agro-systems, microbiome diversity, microbiome isolation and evaluation, microbiome genome annotation, novel mycoviruses in *Foc*, and microbiome product applications.

Cover plants have received considerable attention because of their advantages in increasing the diversity of agro-systems linked to production (Damour et al., 2015). Therefore, the application of cover plants together with resistant cultivars is the best method (Zheng, 2022). In this regard, Wang et al. found that a legume cover plant, Siratro, and natural weeds could change soil fungal diversity in several spans of banana production. They found that fungal diversity significantly decreased after planting bananas. However, both Shannon and Simpson diversity significantly increased after natural weed or legume cover compared to bare soil (Wang et al.). Therefore, the application of suitable cover plants can lead to sustainable banana production by manipulating soil microbial communities.

Soil microbial communities in banana plantations correspond to different ecological conditions. Birt et al. confirmed the core fungal microbiomes of bananas and plantains. There are differences in the diversity and composition of core fungal microbiomes between plant compartments and soils, irrespective of the host genotype (Birt et al.).

Banana-associated effective bacterial and fungal disease suppression is usually achieved through direct isolation from banana plantations. Fan et al. found that *B. velezensis* YN1910 had a significant control effect on FWB. Xiang et al. showed that another *B. velezensis* strain, EB1, also possesses significant antagonistic capability against *Foc* TR4. Yun et al. identified 647 metabolites from the extracts of *S. hygroscopicus* subsp. hygroscopicus 5-4 and found that hygromycin B inhibited the growth rate of *Foc* TR4 mycelia. Different beneficial microbes have specific biological functions. Further investigation into more beneficial microbes adapting to local banana plantations is warranted. Enhancing the biological activities of existing microbes through mutation via traditional physical mutagenesis such as UV and novel physical mutagenesis techniques such as ion beam, intense pulsed light and space mutagenesis could be another direction.

The full genome sequences of biocontrol agents will facilitate the exploitation of specific genes responsible for secondary metabolite production during the development of potential biocontrol agents. In this study, Cuellar-Gaviria et al. reported the complete genome annotation of *B. tequilensis* EA-CB0015. This strain has been shown to efficiently control the banana foliar disease black sigatoka (Cuellar-Gaviria et al.). In the future, more biocontrol agents will be sequenced, and detailed genome annotations will be available. Thus, the whole picture of the genome will shed light on the functional genes involved in the production of effective metabolites and their potential capability.

Mycoviruses from plant pathogens, which induce hypovirulence in host fungi, have received considerable attention as potential biocontrol agents (García-Pedrajas et al., 2019). To explore the presence of mycoviruses in *Foc*, Ye et al. discovered the diversity of *Foc* mycoviruses. They first demonstrated the presence of mycoviruses in *Foc* and discussed the possibility of using mycoviruses for the biocontrol of FWB (Ye et al.). Therefore, the exploration of mycoviruses could be another option for the biocontrol of FWB.

The effective translation of research findings from the laboratory to field applications is essential to confirm the success of biocontrol agents. Tian et al. found that the application of *B. amyloliquefaciens* QST713 significantly affected the diversity of bacteria and fungi in the resistant cultivar Yunjiao No.1 but not in the susceptible cultivar Brazilian. Du et al. designed a compound microbial agent that achieved the most durable control effect in the *Foc* TR4 heavily infected field. The disease control effect reached 57.14% against FWB after the application of the four-strain combination (Du et al.). Damodaran et al. developed an interesting bioimmune system and showed significantly higher weights of bunches, hand numbers per bunch, and finger numbers per hand in treated plants compared to control. Therefore, manipulating banana holobionts by inserting novel endophytes into plantlets to create a new type of “banana hybrid” with increased resilience could be a future direction for enhancing biocontrol agents.

## 3 Conclusion

In the last century, the transition from Gros Michel to Cavendish stands out as the most successful example of employing resistant cultivars for FWB management as a singular solution. However, breeding a new resistant cultivar or transitioning cultivars to meet market demands is not always straightforward. To date, numerous beneficial microbes have been isolated, identified, and evaluated in greenhouse studies; however, their effective application in the field remains limited. Hence, enhancing the colonizing efficiency of the microbe in its host for enduring antagonism against pathogens and unraveling its intricate interaction mechanisms will be imperative for further research. Understanding host-pathogen-endophyte biocontrol interactions is crucial for maintaining sustainable and healthy development in the banana industry, even with *Foc* TR4 present in the long term.

## Author contributions

S-JZ: Writing—review & editing, Writing—original draft, Visualization, Validation, Supervision, Resources, Project administration, Methodology, Investigation, Funding acquisition, Formal analysis, Data curation, Conceptualization. HH: Writing—review & editing, Writing—original draft, Visualization, Resources, Project administration, Conceptualization. YL: Writing—review & editing, Writing—original draft, Visualization, Resources, Project administration, Methodology, Data curation, Conceptualization. JC: Writing—review & editing, Writing—original draft, Visualization, Resources, Project administration, Methodology, Investigation, Conceptualization. XL: Writing—review & editing, Writing—original draft, Visualization, Validation, Resources, Methodology, Investigation, Formal analysis, Conceptualization. TB: Writing—review & editing, Writing—original draft, Visualization, Validation, Resources, Methodology, Investigation, Conceptualization. All authors contributed to the article and approved the submitted version.
